# Two dose levels of rabbit antithymocyte globulin as graft-versus-host disease prophylaxis in haploidentical stem cell transplantation: a multicenter randomized study

**DOI:** 10.1186/s12916-019-1393-7

**Published:** 2019-08-12

**Authors:** Ren Lin, Yu Wang, Fen Huang, Zhiping Fan, Shen Zhang, Ting Yang, Yajing Xu, Na Xu, Li Xuan, Jieyu Ye, Jing Sun, Xiaojun Huang, Qifa Liu

**Affiliations:** 1 Department of Hematology, Nanfang Hospital, Southern Medical University, Guangzhou, China; 2 Department of Hematology, Peking University People’s Hospital, Peking University Institute of Hematology, Beijing, China; 3 Department of Hematology, Fujian Institute of Hematology, Fujian Medical University Union Hospital, Fuzhou, China; 4 Department of Hematology, Xiangya Hospital, Central South University, Changsha, China; 5 Guangdong Provincial Key Laboratory of Construction and Detection in Tissue Engineering, Southern Medical University, Guangzhou, China; 6 Beijing Key Laboratory of Hematopoietic Stem Cell Transplantation, Beijing, China

**Keywords:** Antithymocyte globulin, Haploidentical hematopoietic stem cell transplantation, EBV, CMV, Graft-versus-host disease

## Abstract

**Background:**

The optimal dose of rabbit antithymocyte globulin (ATG, ImtixSangstat) minimizing infections without increasing graft-versus-host disease (GVHD) is unknown in T cell-replete, G-CSF-primed haploidentical hematopoietic stem cell transplantation (haplo-HSCT).

**Methods:**

Four hundred and eight patients were enrolled in this multicenter study to evaluate the effect of 7.5 mg/kg and 10.0 mg/kg rabbit ATG on viral infections and GVHD prophylaxis after haplo-HSCT. The primary endpoint was EBV DNAemia within 1 year posttransplantation.

**Results:**

The 1-year incidence of EBV DNAemia was 20.7% (95% confidence interval, 15.4–26.5) and 40.0% (33.3–46.6) in the 7.5 mg/kg and 10.0 mg/kg groups, respectively (*P* < 0.001). The 100-day cumulative incidence of grade II to IV aGVHD was 27.1% (21.1–33.4) and 25.4% (19.6–31.5) in the 7.5 mg/kg and 10.0 mg/kg ATG groups, respectively (*P* = 0.548). The 2-year incidence of chronic GVHD was 34.6% (27.8–41.4) and 36.2% (29.1–43.2) in the 7.5 mg and 10.0 mg groups (*P* = 0.814). The 1-year incidence of CMV DNAemia was 73.4% (67.2–79.4) and 83.4% (77.5–87.9) in the 7.5 mg/kg and 10.0 mg/kg groups (*P* = 0.038). The 3-year overall survival posttransplantation was 69.5% (63.2–75.8) and 63.5% (56.2–70.8), and the disease-free survival was 62.2% (55.3–69.1) and 60.3% (53.0–67.6) in the 7.5 mg/kg and 10.0 mg/kg groups, respectively (OS: *P* = 0.308; DFS: *P* = 0.660). The counts of EBV- and CMV-specific cytotoxic T cells (CTLs) were higher in the 7.5 mg/kg group than in the 10.0 mg/kg group early posttransplantation.

**Conclusions:**

Compared with 10.0 mg/kg, 7.5 mg/kg ATG for GVHD prophylaxis was associated with reduced EBV and CMV infections without increased incidence of GVHD in haplo-HSCT, probably by affecting EBV- and CMV-specific CTLs.

**Trial registration:**

clinicaltrials.gov, NCT01883180. Registered 14 June 2013.

**Electronic supplementary material:**

The online version of this article (10.1186/s12916-019-1393-7) contains supplementary material, which is available to authorized users.

## Background

Haploidentical related donor transplantation is now considered an important allogeneic hematopoietic stem cell transplantation (allo-HSCT) [1–3]. Currently, the strategies for graft-versus-host disease (GVHD) prophylaxis mainly include ex vivo and in vivo T cell depletion (TCD) in haploidentical HSCT (haplo-HSCT) [4, 5]. In vivo TCD modalities, including antithymocyte globulin (ATG)-based protocols and posttransplantation cyclophosphamide (PTCy) protocols, have been widely used [5–8]. The ATG strategy has become mainstream in Asia including China, Japan, and Korea [1, 2, 9]. Nevertheless, infections, especially viral infections, remain an important drawback of this strategy [5, 6].

To date, the optimal dose of ATG that has sufficient efficacy for GVHD prophylaxis and minimizes the risk of infection is unknown for haplo-HSCT. Several studies have demonstrated that the risk of infection depends on the dose of ATG [6, 10, 11]. In our previous single-center study, 6 mg/kg rabbit ATG was associated with a lower incidence of Epstein-Barr virus (EBV) infection but higher risks of severe acute GVHD (aGVHD) and chronic GVHD (cGVHD) in haplo-HSCT than 10 mg/kg rabbit ATG [10, 12]. As the decreased mortality from non-GVHD complications in the 6 mg/kg ATG group was offset by an increased incidence of severe GVHD, the optimal dose was left undetermined [10]. A middle dose of 7.5 mg/kg ATG, which is the common dose used for unrelated HSCT, needs to be evaluated in haplo-HSCT. Here, we designed a multicenter randomized study to evaluate the effect of 7.5 mg/kg and 10.0 mg/kg rabbit ATG aiming at reducing the incidence of viral infections without increasing the incidence of GVHD in haplo-HSCT.

## Methods

### Study design and randomization

This multicenter, open-label, randomized study was performed in four hospitals in China including Nanfang Hospital, Peking University People’s Hospital, Fujian Medical University Union Hospital, and Xiangya Hospital. The study protocol is shown in Additional file 1: Study protocol. The study protocol was approved by the local ethics committee review board, and written informed consent was obtained from the donors and recipients in accordance with the Declaration of Helsinki before the initiation of the study. Patients were randomly assigned to the 7.5 mg/kg or the 10.0 mg/kg ATG group in a 1:1 ratio. Randomization was performed with randomization codes generated by computer-generated randomization system, and patients were stratified by the transplant center.

### Patients and eligibility criteria

Patients with acute leukemia who were scheduled to receive haplo-HSCT were eligible for the study if they were 14 to 65 years old. Haplo-HSCT was administered if a suitable human leukocyte antigen (HLA)-matched sibling donor or a suitable HLA-matched unrelated donor was unavailable within the timeframe appropriate for the patient’s clinical circumstances; donor selection and HLA typing were performed as described previously [13]. Patients were excluded if they had existing contraindications for HSCT or a known hypersensitivity to ATG. In addition, patients or donors who had active cytomegalovirus (CMV) or EBV infections (viremia or diseases) at the time of transplantation were excluded.

### Conditioning and transplants

Two conditioning regimens were used for transplants: modified busulfan/cyclophosphamide (mBuCy) and a sequential intensified regimen. The mBuCy regimen included cytarabine (Ara-C) (4 g/m^2^/day, days − 10 and − 9), Bu (3.2 mg/kg/day, days − 8 to − 6), Cy (1.8 g/m^2^/kg, days − 5 and − 4), and simustine (250 mg/m^2^, day − 3). The sequential intensified regimen consisted of fludarabine (30 mg/kg/day, days − 10 to − 6), Ara-C (2 g/m^2^/day, days − 10 to − 6), total body irradiation (TBI, 4.5 Gy/day, days − 5 and − 4), Cy (60 mg/kg/day, days − 3 and − 2), and etoposide (15 mg/kg/day, days − 3 and − 2). The selection of the conditioning regimen was based on disease status at transplantation. The patients in complete remission (CR) received the mBuCy regimen, while those in non-CR received the sequential intensified regimen [14]. G-CSF mobilized fresh bone marrow and peripheral blood stem cell grafts were infused [2].

### GVHD prophylaxis

Short-term methotrexate (15 mg/m^2^, on days + 1, + 3, and + 6), cyclosporin A (CsA), and mycophenolate (MMF) were used for GVHD prophylaxis in all the patients. ATG (rabbit anti-human thymocyte immunoglobulin, ImtixSangstat, Lyon, France) was administered at 2.5 mg/kg/day from days − 3 to − 1 in the 7.5 mg/kg group or days − 4 to − 1 in the 10.0 mg/kg group. If diarrhea or grade III fever (according to the Common Terminology Criteria for Adverse Events) occurred, dexamethasone (5.0 mg) was administered for treatment and the speed of the ATG infusion was decreased. CsA and MMF were administered as described previously [2]. The primary dosage of CsA was 2.5 mg/kg/day i.v. from day − 9 until bowel function returned to normal. At that point, the patient was switched to oral CsA. The dosage was adjusted to maintain a blood trough concentration of 200–300 ng/mL. MMF was administered at 0.5 g every 12 h from day − 9 to day + 30, tapered to 0.25 g every 12 h on day + 30, and discontinued over days + 45 to + 60.

### CMV-DNA and EBV-DNA monitoring

As described previously [15], the EBV- and CMV-DNA loads in the blood were measured regularly by real-time quantitative polymerase chain reaction (RQ-PCR). The threshold for EBV-DNA and CMV-DNA copies in plasma provided by the manufacturer (ZJ Bio-Tech Co., Ltd., Shanghai, China) was less than 500 copies/ml. The EBV- and CMV-DNA loads in the blood were monitored weekly for the first 3 months after transplantation, once every 2 weeks from the 4th to the 9th month posttransplantation and then once per month from the 10th to the 12th month. Once EBV-DNA or CMV-DNA in the blood was positive, the viral loads would be detected once again the next day. If positive, viral loads were monitored twice a week.

### Pre-emptive therapy for EBV and CMV DNAemia

Pre-emptive therapy was given to the patients who developed viremia without a sign of viral diseases. For the pre-emptive therapy of EBV DNAemia, rituximab was used based on EBV-DNA loads and durative time of EBV DNAemia. Rituximab-based preemptive therapy was administrated as follows: (1) EBV-DNA in blood was positive twice consecutively with a rising trend over 1 log; (2) EBV-DNA was positive consecutively 6 times without a dropping trend over 1 log; or (3) EBV-DNA was positive consecutively 8 times. Rituximab preemptive therapy (375 mg/m^2^) was given once weekly and a total of 4 doses was 1 cycle. The interval between 2 cycles was 2 weeks. Rituximab was discontinued when EBV-DNA turned negative consecutively 4 times. To the patients who showed non-complete response after 1 cycle of rituximab treatment, the adoptive cellular immunotherapies following rituximab, including donor lymphocyte infusion (DLI) or EBV-specific cytotoxic T lymphocytes (EBV-CTL), were used as described previously [16, 17]. For the preemptive therapy of CMV DNAemia, ganciclovir was used until CMV-DNA turned negative consecutively 2 times. Foscarnet was an alternative to ganciclovir in case of cytopenia. Immunosuppressive drugs were reduced whenever feasible to all patients.

### Immune reconstitution

Immune reconstitution was assessed as previously described [15]. EBV- and CMV -specific cytotoxic T cell (CTL) reconstitutions were tested at the 1st, 2nd, 3rd, and 6th months posttransplantation in consecutive patients undergoing haplo-HSCT whose HLA-A type was 0201 or 2402 using an enzyme-linked immunospot assay [18, 19].

### Evaluation points and definition

The primary endpoint was EBV DNAemia within 1 year posttransplantation. EBV DNAemia was defined as EBV-DNA in blood positive twice consecutively. Once EBV-DNA in the blood was positive, the viral loads would be detected once again the next day. The secondary endpoints included aGVHD, cGVHD, CMV DNAemia, EBV- and CMV-associated diseases, engraftment, leukemia relapse, nonrelapse mortality (NRM), overall survival (OS), disease-free survival (DFS), and tolerability. aGVHD was defined according to the 1994 Consensus Conference on Acute GVHD Grading and graded from I to IV [20], and cGVHD was graded as limited or extensive according to the literature [21]. The diagnoses of EBV- and CMV-associated diseases were made according to the guideline [22] and our previous description [15]. Engraftment, primary poor graft function (PGF), relapse, NRM, OS, and DFS were assessed as previously described [2]. GVHD-free and relapse-free survival (GRFS) was defined as patients without grades III to IV acute GVHD, without systemic immunosuppression for chronic GVHD, and surviving without relapse.

Independent clinical monitoring was performed regularly by a data and safety monitoring committee composed of hematologists. To ensure data uniformity, this committee was blinded as to the patients’ treatment assignments, and the data recorded in the case report forms were verified by inspection of the source data in the patients’ charts. To guarantee a correct and consistent assessment of GVHD, detailed structured data of aGVHD and cGVHD were recorded in the case report form and then were reviewed and classified regularly by the data and safety monitoring committee.

### Statistical analysis

In the initial design, we predefined grade II to IV acute GVHD as the primary endpoint. We hypothesized that non-inferiority of 7.5 mg/kg ATG against 10.0 mg/kg ATG was established if the difference of the 95% CI in grade II to IV acute GVHD between the two groups was within 15%. After the analysis of the first 210 patients enrolled in the study, the 100-day incidence of grade II to IV acute GVHD was 28.9% and 27.8% in the 7.5 mg/kg and 10.0 mg/kg groups with a difference of 1.2% with 95% CI of − 10.0 to 13.4%. The 1-year incidence of EBV DNAemia in the 7.5 mg/kg group was significantly lower than that in the 10.0 mg/kg group (27.1% in 7.5 mg/kg group vs 45.2% in the 10.0 mg/kg group). According to the recommendation of the data and safety monitoring committee, the change of the primary endpoint was made to evaluate the superiority of a dose level of 7.5 mg ATG on viral infections without increasing aGVHD after haplo-HSCT. A planned sample size of 197 patients per arm was required for a power of 80% against the hypothesis of a 12% absolute reduction from 42% of patients developing EBV DNAemia (significance level, *P* = 0.05). Considering 5% loss of patients, it was decided to enroll 412 patients (206 in each group). The *χ*^2^ test and Mann-Whitney *U* test were used for categorical variables and continuous variables, respectively. OS and DFS were estimated using the Kaplan-Meier method. NRM, relapse, GVHD, and viral infections were estimated as cumulative incidences, taking into account competing risks. Competing events were defined as follows: for NRM, relapse; for aGVHD, death without aGVHD and relapse; for cGVHD, death without cGVHD and relapse; for EBV DNAemia/PTLD, death without EBV DNAemia/PTLD; and for CMV DNAemia/associated diseases, death without CMV DNAemia/associated diseases. A Cox proportional hazards model was used to evaluate the associations of patient and transplant characteristics with outcomes in a multivariate analysis. All *P* values are based on two-sided hypothesis tests. Alpha was set at 0.05. SPSS 19.0 (SPSS Inc., Chicago, IL, USA) and R version 3.4.3 (R Development Core Team, Vienna, Austria) were used for data analysis. Our data were analyzed on September 30, 2017. The study was registered at clinicaltrials.gov (NCT01883180).

## Results

### Study population

**Fig. 1:**
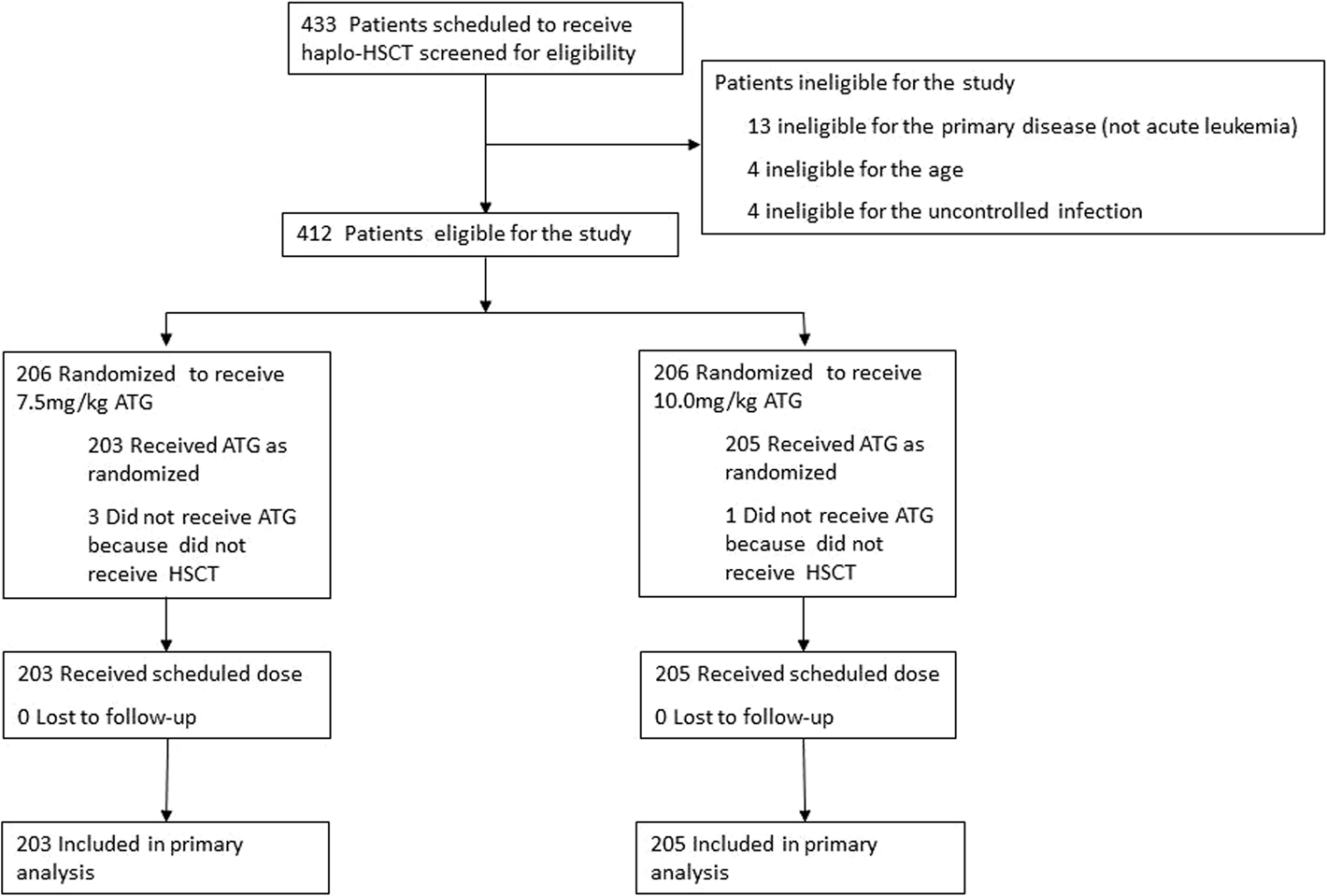
Consolidated Standards of Reporting Trials (CONSORT) diagram

Between June 2013 and January 2016, 412 consecutive patients were enrolled and randomized in this study. Two hundred and six patients were randomized to the 7.5 mg/kg group, and 206 to the 10.0 mg/kg group. Three patients in the 7.5 mg/kg group and one in the 10.0 mg/kg group did not receive allocated intervention and transplantation due to leukemia relapse before transplant or toxicity caused by the conditioning regimens. Therefore, 203 patients in the 7.5 mg/kg group and 205 in the 10.0 mg/kg group completed this study. A total of 162 patients in Nanfang Hospital were eligible for this study, and 160 were treated (2 did not receive allocated intervention and transplantation due to leukemia relapse before transplant). One hundred and forty-four patients were eligible for this study and treated in Peking University People’s Hospital. Fifty-six were eligible for this study in Fujian Medical University Union Hospital, and 54 were treated (1 did not receive allocated intervention and transplantation due to leukemia relapse before transplant and 1 due to toxicity caused by the conditioning regimens). Fifty were eligible for this study and treated in Xiangya Hospital. The study flow diagram is shown in Fig. 1. The patient and transplant characteristics were balanced between the two groups (Table 1).

**Table 1:** Patient and transplant characteristics

	7.5 mg group (*N* = 203)	10.0 mg group (*N* = 205)	*P*
Patient age, median (range), years	28 (14–59)	26 (14–57)	0.324
Gender, male/female, *n* (%)	139 (68.5)/64 (31.5)	139 (67.5)/66 (32.2)	0.885
Primary diseases, *n* (%)			0.483
AML (cytogenetics)	99 (48.8)	95 (46.3)	
Better risk	8 (8.1)	7 (7.4)	
Intermediate risk	41 (41.4)	41 (43.1)	
Poor risk	36 (36.4)	32 (33.7)	
Unknown	14 (14.1)	15 (15.8)	
ALL	94 (46.3)	94 (45.6)	
Ph positive	29 (30.9)	31 (33.0)	
Ph negative	65 (69.1)	63 (67.0)	
ABL/ALAL	10 (4.9)	16 (7.8)	
Number of courses of chemotherapy before transplantation, median (range)	3 (3–9)	3 (3–7)	0.875
Status of primary disease, *n* (%)			0.318
CR1	155 (76.4)	143 (69.7)	
≥ CR2	10 (4.9)	12 (5.9)	
Non-CR	38 (18.7)	50 (24.4)	
Conditioning, *n* (%)			0.164
Standard myeloablative regimen	165 (81.3)	155 (75.6)	
Intensified conditioning	38 (18.7)	50 (24.3)	
No. of HLA mismatched, *n* (%)			0.479
1	14 (6.9)	15 (7.3)	
2–3	51 (25.1)	62 (30.2)	
4–5	138 (68.0)	128 (62.1)	
Donor age, median (range), years	38 (10–59)	40 (15–59)	0.326
Donor–recipient sex match, *n* (%)			0.850
Male–male	90 (44.3)	92 (44.9)	
Male–female	41 (20.2)	47 (22.9)	
Female–male	55 (27.1)	52 (25.4)	
Female–female	17 (8.4)	14 (6.8)	
CMV serostatus, *n* (%)			0.906
D−/R−	8 (3.9)	10 (4.9)	
D+/R−	7 (3.4)	7 (3.4)	
D−/R+	5 (2.5)	7 (3.4)	
D+/R+	183 (90.1)	181 (88.3)	
EBV serostatus, *n* (%)			0.940
D−/R−	9 (4.4)	10 (4.9)	
D+/R−	47 (23.2)	50 (24.3)	
D−/R+	34 (16.7)	30 (14.6)	
D+/R+	113 (55.7)	115 (56.1)	
Median CD34+ cells per graft, × 10^6^/kg (range)	2.26 (0.32–7.37)	2.58 (0.71–7.39)	0.205

*AML* acute myeloid leukemia, *ALL* acute lymphoblastic leukemia, *ABL* acute biphenotypic leukemia, *ALAL* acute leukemia of ambiguous lineage, *CR* complete remission

### Engraftment

All 203 patients in the 7.5 mg/kg group achieved engraftment within 30 days posttransplantation except one who developed primary PGF and died of infection on days + 75. Of the 205 patients in the 10.0 mg/kg group, 202 achieved engraftment within 30 days posttransplantation, while 3 died of infections within 2 weeks posttransplantation, and 1 developed primary PGF and died of infection on days + 85. The median time to neutrophil reconstitution was 12 (9–45) days and 13 (10–43) days in the 7.5 mg/kg and 10.0 mg/kg groups, respectively (*P* = 0.305). The median platelet reconstitution time was 13 (8–119) days and 15 (7–160) days in the 7.5 mg/kg and 10.0 mg/kg groups, respectively (*P* = 0.138).

### EBV and CMV infections

**Fig. 2:**
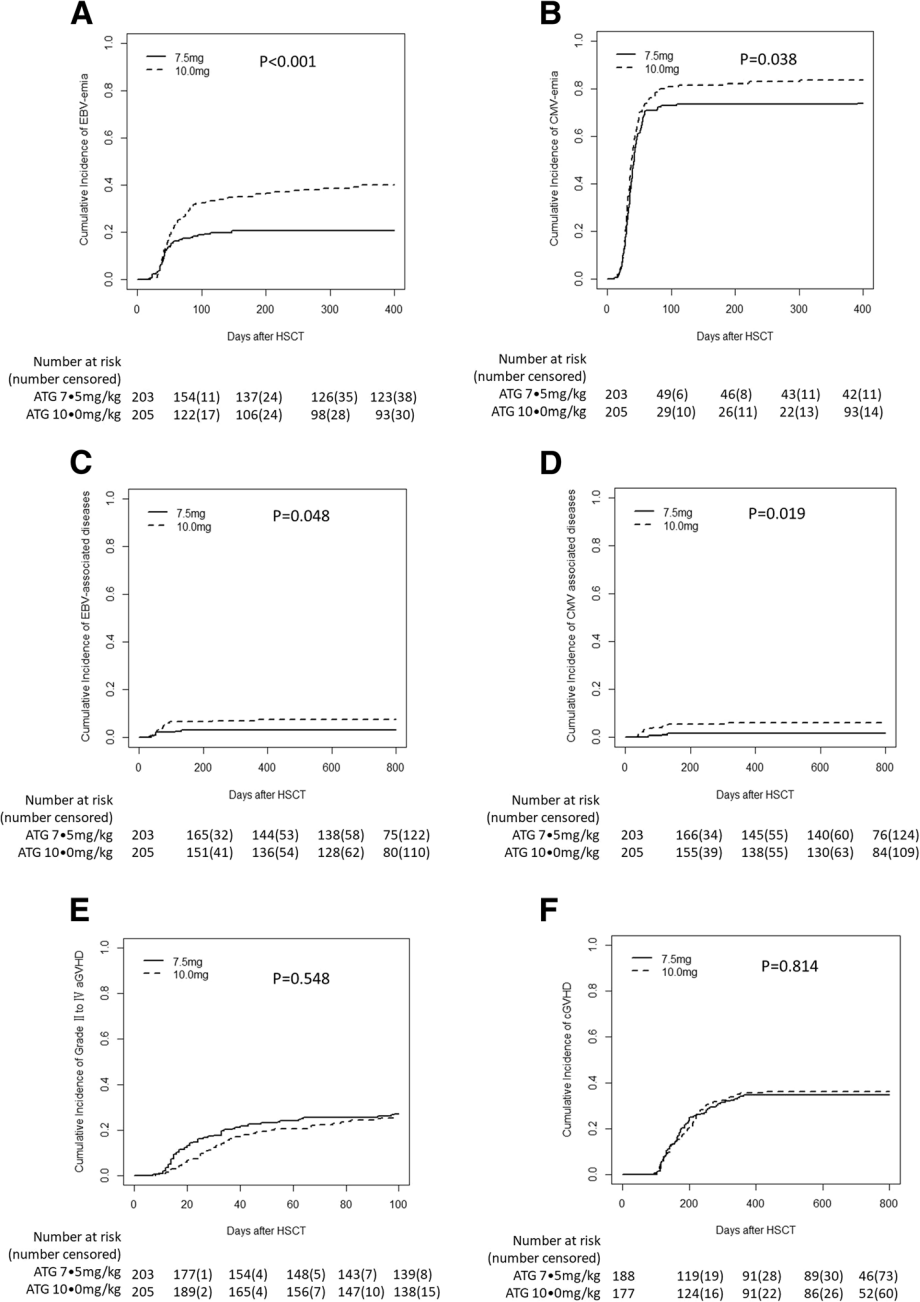
Outcomes of haplo-HSCT in the two groups. **a** EBV DNAemia. **b** CMV DNAemia. **c** EBV-associated diseases. **d** CMV-associated diseases. **e** Grades II to IV acute GVHD (aGVHD). **f** Overall chronic GVHD (cGVHD)

The 1-year cumulative incidence of EBV DNAemia was 20.7% [95% confidence interval (CI), 15.4–26.5%] and 40.0% (33.3–46.6%) in the 7.5 mg/kg and 10.0 mg/kg groups, respectively (*P* < 0.001, Fig. 2a). No difference in the incidence of EBV DNAemia was documented among the four centers (*P* = 0.237). Among the first 210 patients enrolled in the study, the 1-year incidence of EBV DNAemia was 27.1% and 45.2% in the 7.5 mg/kg and 10.0 mg/kg groups (*P* < 0.01). For the rest, 198 patients enrolled in this study; 1-year incidence of EBV DNAemia was 13.7% and 33.3% in the 7.5 mg/kg and 10.0 mg/kg groups (*P* < 0.01). The incidence of EBV DNAemia was significantly lower in the 7.5 mg/kg group than that in 10.0 mg/kg group both in the cohort of the first 210 patients and the rest 198 patients. The possible cause of the differences in the incidence of EBV DNAemia between the two cohorts was further analyzed. More patients were at NR status of primary disease at the time of transplant and received the intensified conditioning (the conditioning regimen was chosen according to the study design) in the first cohort than those in the second cohort (*P* < 0.01). The intensified conditioning is positive related to the EBV DNAemia in the two cohorts (both *P* < 0.01). The 1-year cumulative incidence of CMV DNAemia was 73.4% (67.2–79.4%) and 83.4% (77.5–87.9%) in the 7.5 mg/kg and 10.0 mg/kg groups, respectively (*P* = 0.038, Fig. 2b). More patients with EBV DNAemia received rituximab preemptive therapy in the 10.0 mg/kg group than those in 7.5 mg/kg group (24.4% vs 13.8%, *P* = 0.006). With the median follow-up of 707 (range, 1–1582) days posttransplantation, 6 patients developed EBV-associated diseases in the 7.5 mg/kg group [4 with posttransplant lymphoproliferative disorder (PTLD) and 2 with encephalitis], and 15 patients in the 10.0 mg/kg group developed EBV-associated diseases (15 with PTLD). The 2-year cumulative incidence of EBV-associated diseases posttransplantation was 3.0% (1.2–6.0%) and 7.3% (4.3–11.4%) in the 7.5 mg/kg and 10.0 mg/kg groups, respectively (*P* = 0.048, Fig. 2c). Two patients died of PTLD in the 10.0 mg/kg group, while none in the 7.5 mg/kg group died (*P* = 0.499). Three patients developed CMV-associated diseases (2 with pneumonia and 1 with retinitis) in the 7.5 mg/kg group and 12 (8 with pneumonia, 2 with retinitis, and 2 with enteritis) in the 10.0 mg/kg group. The 2-year incidence of CMV-associated diseases was 1.5% (0.4–4.0%) and 5.9% (3.2–9.7%) in the 7.5 mg/kg and 10.0 mg/kg groups, respectively (*P* = 0.019, Fig. 2d). Six patients died of CMV-associated diseases, including 1 in the 7.5 mg/kg group and 5 in the 10.0 mg/kg group (*P* = 0.215). The risk factors for EBV and CMV infections are shown in Table 2. A 10.0-mg/kg dose of ATG was an independent risk factor for EBV and CMV DNAemia as well as CMV-associated diseases posttransplantation.

**Table 2:** Multivariate analysis of risk factors for EBV and CMV infections

Risk factors	EBV DNAemia		EBV-associated disease		CMV DNAemia		CMV-associated diseases	
	HR(95%CI)	*P*	HR, 95%CI	*P*	HR, 95%CI	P	HR, 95%CI	*P*
Patient gender								
Male	1	0.084	1	0.110	1	0.140	1	0.560
Female	0.70 (0.47–1.05)		0.35(0.10–1.26)		1.18 (0.95–1.48)		0.71 (0.23–2.23)	
Patient age								
**<** 27 years	1	0.570	1	0.300	*1*	*0.003*	1	0.470
≥ 27 years	0.90 (0.63–1.29)		1.58,(0.67–3.74)		*1.39 (1.11–1.73)*		0.69 (0.24–1.93)	
Conditioning								
BuCy	*1*	*0.005*	*1*	0.061	1	0.380	1	0.420
Intensified	*1.73 (1.18–2.54)*		*2.28 (0.96–5.42)*		0.88 (0.67–1.17)		1.55 (0.54–4.45)	
EBV serostatus,					–		–	
Other	1	0.046	*1*	*0.038*		–		–
D−/R+	1.58 (1.01–2.46)		*2.85 (1.06–7.670*					
CMV serostatus	–		–					
Other		–		–	1	0.420	1	0.051
D−/R+					0.79 (0.44–1.41)		4.54 (0.99–20.78)	
ATG dose,								
7.5 mg/kg	*1*	*< 0.001*	*1*	0.065	*1*	*0.013*	*1*	*0.050*
10.0 mg/kg	*2.02 (1.37–2.97)*		*2.45 (0.95–6.33)*		*1.31 (1.06–1.62)*		*3.65 (1.00–13.32)*	

*HR* hazard ratio

### aGVHD and cGVHD

The 100-day cumulative incidence of grade II to IV aGVHD was 27.1% (21.1–33.4%) and 25.4% (19.6–31.5%) in the 7.5 mg/kg and 10.0 mg/kg ATG groups, respectively (*P* = 0.548, Fig. 2e). No difference in the incidence of grade II to IV acute GVHD was observed among the four centers (*P* = 0.874). The 100-day cumulative incidence of grade III to IV aGVHD was 7.9% (4.7–12.2%) and 5.4% (2.8–9.1%) in the 7.5 mg/kg and 10.0 mg/kg groups, respectively (*P* = 0.299). Of the 365 patients who survived> 100 days posttransplantation, the 2-year cumulative overall incidence of cGVHD was 34.6% (27.8–41.4%) and 36.2% (29.1–43.2%) in the 7.5 mg and 10.0 mg groups, respectively (*P* = 0.814, Fig. 2f). The 2-year cumulative incidences of limited and extensive cGVHD were 21.8% (16.2–28.0%) and 12.8% (8.5–18.0%), respectively, in the 7.5 mg/kg group and 26.0% (19.8–32.6%) and 10.2% (6.3–15.2%), respectively, in the 10.0 mg/kg group (limited: *P* = 0.354; extensive: *P* = 0.417). The risk factors for aGVHD and cGVHD are shown in Additional file 2: Table S1.

### Immune reconstitution

Immune reconstitution was similar with respect to the counts and percentages of T cell subsets, B cells, and NK cells between the two groups (Additional file 2: Table S2 and S3). Between February 2015 and June 2015, EBV- and CMV-CTLs were detected in 31 patients (13 in the 7.5 mg/kg group and 18 in the 10.0 mg/kg group) at the 1st, 2nd, 3rd, and 6th months posttransplantation. The counts of EBV- and CMV-specific CTLs were higher in the 7.5 mg/kg group than in the 10.0 mg/kg group during the 2nd month posttransplantation (EBV: *P* = 0.046; CMV: *P* = 0.050; Table 3).

**Table 3:** Reconstitution of EBV-CTLs and CMV-CTLs within 6 months posttransplantation

Months after transplantation	EBV-CTLs (spots/10^5^ cells)			CMV-CTLs (spots/10^5^ cells)		
	7.5 mg/kg group, mean counts (range)	10.0 mg/kg group, mean counts (range)	*P*	7.5 mg/kg group, mean counts (range)	10.0 mg/kg group, mean counts (range)	*P*
1st	50.08 (23–136)	38.61 (16–110)	0.249	57.38 (20–163)	45.56 (19–131)	0.312
2nd	89.23 (34–254)	47.28 (20–173)	*0.046*	131.08 (75–465)	69.72 (21–311)	0.050
3rd	166.08 (45–528)	97.17 (26–364)	0.189	167.08 (30–501)	110.67 (29–399)	0.229
6th	122.38 (38–458)	108.94 (24–453)	0.752	84.38 (35–258)	75.44 (24–301)	0.698

*CTLs* specific cytotoxic T cells

### Tolerability

All the patients suffered fever during ATG infusion, and glucocorticoids were effective. The incidence of gastrointestinal symptoms was 53.2% and 58.0% in the 7.5 mg/kg and 10.0 mg/kg groups, respectively (*P* = 0.324). The main adverse event associated with ATG was a serum sickness-like reaction (13.0%). All the patients completed the ATG intervention.

### Survival and relapse

**Fig. 3:**
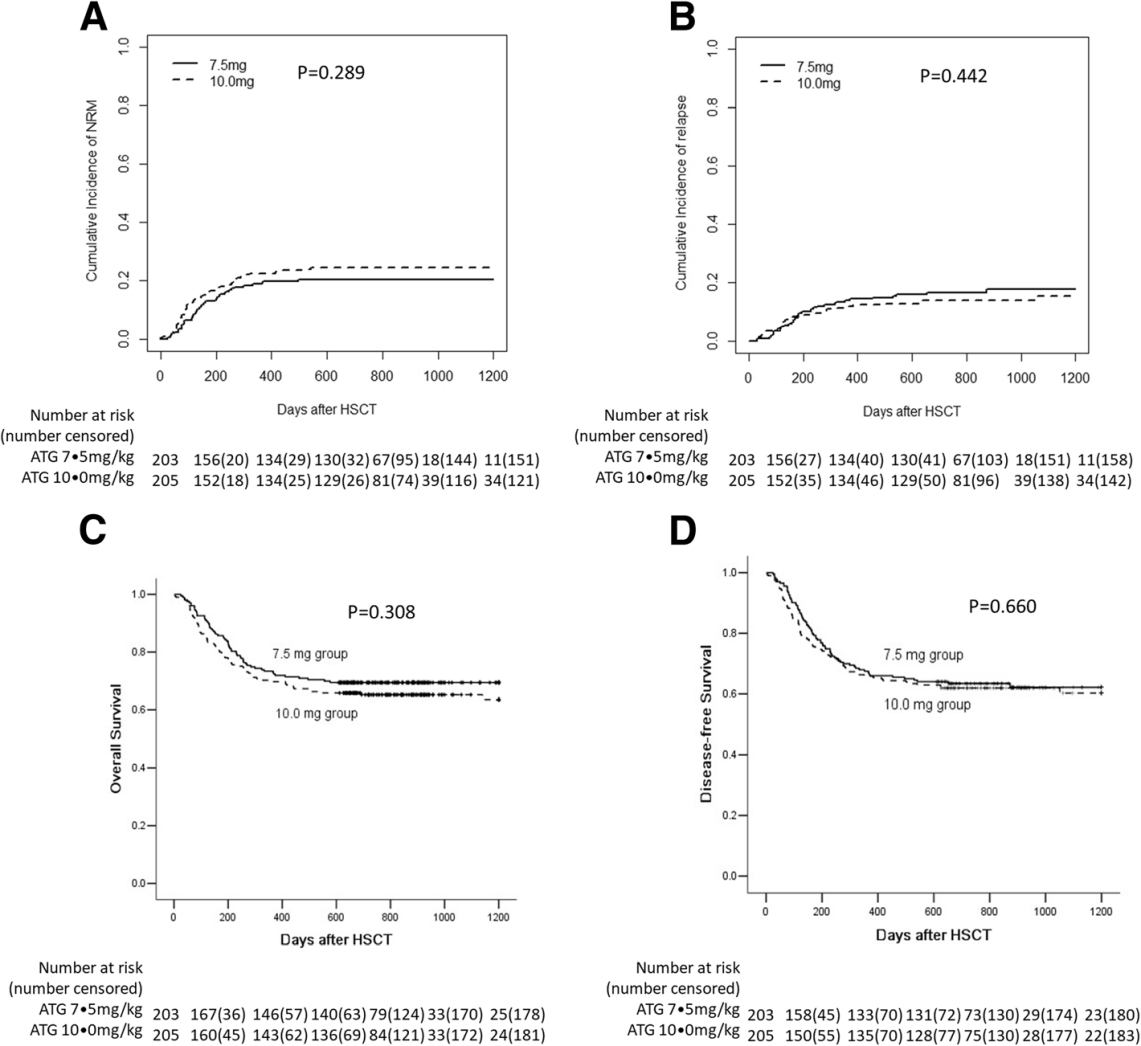
Survival and relapse in the two groups. **a** Nonrelapse mortality (NRM). **b** Leukemia relapse. **c** Overall survival (OS). **d** Disease-free survival (DFS)

Within the follow-up period, 274 patients survived and 134 died (62 in the 7.5 mg/kg group and 72 in the 10.0 mg/kg group). The causes of death are presented in Additional file 2: Table S4. There were no differences in GVHD-related deaths (*P* = 0.556) or infection-related deaths (*P* = 0.393) between the two groups. The 3-year cumulative incidence of NRM was 20.2% (15.0–26.0%) and 24.4% (18.7–30.4%) in the 7.5 mg/kg and 10.0 mg/kg groups, respectively (*P* = 0.289, Fig. 3a). The 3-year incidence of relapse posttransplantation was similar between the two groups [17.6% (12.4–23.6%) vs 15.3% (10.2–21.5%); *P* = 0.442, Fig. 3b]. The 3-year OS was 69.5% (63.2–75.8%) and 63.5% (56.2–70.8%) in the 7.5 mg/kg and 10 mg/kg groups, respectively, and the DFS was 62.2% (55.3–69.1%) and 60.3% (53.0–67.6%) in the 7.5 mg/kg and 10.0 mg/kg groups, respectively (OS: *P* = 0.308, Fig. 3c; DFS: *P* = 0.660, Fig. 3d). No difference in OS was documented among the four centers (*P* = 0.538). The 3-year GRFS was 36.0% (29.4–42.6) and 31.7% (25.2–42.6) in the 7.5 mg/kg and 10.0 mg/kg groups, respectively (*P* = 0.544). The risk factors for survival and relapse are presented in Additional file 2: Table S5.

## Discussion

At present, the optimal dose of ATG for GVHD prophylaxis minimizing risk of infections without increasing GVHD in a haplo-HSCT setting remains undefined [6, 10]. In this study, we have explored the effects of two dose levels of ATG on viral infections and GVHD for patients undergoing in vivo TCD transplantation from haploidentical donors. EBV DNAemia was the primary endpoint, and GVHD was the secondary endpoint. Our results showed that, compared to 10.0 mg/kg ATG, 7.5 mg/kg ATG was associated with a significant reduced risk of EBV and CMV infections, while the incidence of GVHD was similar between the two dose levels. To the best of our knowledge, this is the first multicenter randomized controlled trial to investigate the preferred dose of ATG for GVHD prophylaxis in a haplo-HSCT setting.

The dose of ATG was positively related to the incidences of infections and negatively related to aGVHD in transplant recipients [15, 23–25]. Several studies have suggested that a total dose of 4.5–5 mg/kg and 7.5 mg/kg rabbit ATG are preferred for HLA-matched sibling donor and unrelated donor transplantation, respectively, and these doses provide adequate GVHD prevention without increasing infections [25–29]. However, most of these studies were not randomized controlled trial. An Italian group conducted a multicenter randomized controlled trial to compare 2 different doses (30 mg/kg vs 15 mg/kg) of rabbit anti-T-lymphocyte globulin (ATLG), and the results showed that a lower dose resulted in improved OS and EFS [30]. In the haplo-HSCT setting, the optimal dose of ATG balancing the efficacy of GVHD prophylaxis and the risk of infection is lacking. Our previous single-center prospective study documented that 6.0 mg/kg dose of ATG was associated with a lower incidence of EBV DNAemia but a higher incidence of aGVHD and cGVHD than a 10.0-mg/kg dose in a haploidentical setting [10]. In the current study, we found the patients receiving 7.5 mg/kg ATG had a lower incidence of EBV and CMV DNAemia and a similar incidence of aGVHD and cGVHD compared with those receiving 10.0 mg/kg. In addition, the multivariate analysis revealed that the higher dose of ATG was a risk factor for EBV and CMV infections. This result met the primary endpoint of the study. The delayed immune reconstitution posttransplantation caused by ATG was considered an important risk factor for viral infections [11, 31]. Duval et al. [32] reported slower T cells and B cells reconstitution in patients treated with high-dose ATG (14.4 to 19.4 mg/kg) than in those with low-dose ATG (2.5 to 10.5 mg/kg) during HLA-matched unrelated donor transplantation or haplo-HSCT. Our previous study also revealed that the patients treated with a 6.0-mg/kg dose of ATG had faster T cell reconstitution than those with a 10.0-mg/kg dose during the first 2 months of haplo-HSCT [31]. In this study, although the reconstitution of T cell subsets, B cells, and NK cells were similar between the two groups, better reconstitution of CMV- and EBV-CTLs was documented in the 7.5 mg/kg group than in the 10.0 mg/kg group, which might be the reason for the lower incidence of viral infections in the 7.5 mg/kg group. Based on these results, 7.5 mg/kg ATG seems to be more appropriate for balancing infection control and GVHD prophylaxis.

Considering the association between delayed immune reconstitution and ATG dose, whether a high dose of ATG might increase the incidence of leukemia relapse is still an unresolved matter. In haplo-HSCT, reports regarding the effect of ATG dose on relapse are also lacking [10, 12]. The results of our single-center study showed that the 5-year incidence of relapse was comparable between the 6.0 mg/kg and 10.0 mg/kg groups in haplo-HSCT setting [12]. In this study, the incidence of relapse was not different between the 7.5 mg/kg and 10.0 mg/kg groups.

A beneficial effect of NRM was not seen although 7.5 mg/kg ATG was associated with lower incidences of EBV and CMV infections and the comparable morbidity and mortality associated with GVHD. A reasonable interpretation of these findings is the similar mortality of viral diseases between the two groups, which were attributed to the effective therapy for viral diseases.

Compared with those patients with 10.0 mg/kg ATG, fewer patients underwent therapies for EBV and CMV DNAemia as well as associated diseases although survival benefit has not been observed. This might bring a potential economic benefit in the patients receiving 7.5 mg/kg ATG and therefore less side effects of pre-emptive therapies.

A limitation of this study was the limited sample size of patients of whom the reconstitution of EBV- and CMV-CTLs posttransplantation was assessed. More patients should be enrolled in further study to explore the effect of ATG on EBV- and CMV-CTL reconstitution.

## Conclusions

Compared with 10.0 mg/kg, 7.5 mg/kg ATG as GVHD prophylaxis is associated with a reduced incidence of EBV and CMV infections during haplo-HSCT without increasing the incidence and mortality associated with GVHD, probably by affecting EBV- and CMV-CTLs. If replicated, these findings favor the use of 7.5 mg/kg ATG in patients undergoing T cell-replete, G-CSF-primed haplo-HSCT.

## Additional files

Additional file 1: Study protocol. (DOCX 60 kb)

Additional file 2: **Table S1.** Risk factors for aGVHD and cGVHD: Including univariate and multivariate risk factors analysis for aGVHD and cGVHD. **Table S2.** Immune reconstitution within 1 year posttransplantation (absolute value): Including the T, B, and NK cells reconstitution within 1 year posttransplantation shown by the absolute value. **Table S3.** Immune reconstitution within 1 year posttransplantation (percentage): Including the T, B, and NK cells reconstitution within 1 year posttransplantation shown by the percentage. **Table S4.** Causes of death posttransplantation. **Table S5.** Risk factors for survival and relapse: Including univariate and multivariate risk factors analysis for survival and relapse. (DOCX 51 kb)

## Data Availability

Technical appendix, statistical code, and dataset available from the corresponding author at liuqifa628@163.com.

## Abbreviations

aGVHD: Acute GVHD
allo-HSCT: Allogeneic hematopoietic stem cell transplantation
Ara-C: Cytarabine
ATG: Antithymocyte globulin
cGVHD: Chronic GVHD
CMV: Cytomegalovirus
CR: Complete remission
CsA: Cyclosporin A
DFS: Disease-free survival
DLI: Donor lymphocyte infusion
EBV: Epstein-Barr virus
EBV-CTL: EBV-specific cytotoxic T lymphocytes
G-CSF: Granulocyte colony-stimulating factor
GVHD: Graft-versus-host disease
haplo-HSCT: Haploidentical hematopoietic stem cell transplantation
HLA: Human leukocyte antigen
mBuCy: Modified busulfan/cyclophosphamide
MMF: Mycophenolate
NRM: Nonrelapse mortality
OS: Overall survival
PGF: Poor graft function
PTCy: Posttransplantation cyclophosphamide
PTLD: Posttransplant lymphoproliferative disorder
RQ-PCR: Real-time quantitative polymerase chain reaction
TCD: T cell depletion
