# Supplementary material for: Two dose levels of rabbit antithymocyte globulin as graft-versus-host disease prophylaxis in haploidentical stem cell transplantation: a multicenter randomized study
Source: BMC Med. 2019 Aug 12;17:156. doi: 10.1186/s12916-019-1393-7 (PMC6689871; doi:10.1186/s12916-019-1393-7)
Supplement: Supplementary file 1 — Study protocol. (DOCX 60 kb) [file 12916_2019_1393_MOESM1_ESM.docx]

Protocol of the study

A prospective, randomized, controlled, multi-center study to compare the different doses of ATG used in haploidentical hematopoietic stem cell transplantation

Applicant Institution: Nanfang Hospital, Southern Medical University

Principal institution: Nanfang Hospital, Southern Medical University

Table of Contents

[Protocol Summary 4](#_Toc512010108)

[1. Background 6](#_Toc512010109)

[2. Objectives 7](#_Toc512010110)

[2.1 Primary Objective 7](#_Toc512010111)

[2.2 Secondary Objective 7](#_Toc512010112)

[3. Study Design 7](#_Toc512010113)

[4. Subject Selection Criteria 8](#_Toc512010114)

[4.1 Subject Selection Criteria 8](#_Toc512010115)

[4.1.1 Number of Subjects 8](#_Toc512010116)

[4.1.2 Inclusion Criteria 8](#_Toc512010117)

[4.1.3 Exclusion Criteria 8](#_Toc512010118)

[4.2. Removal Criteria 9](#_Toc512010119)

[4.3. Withdrawal Criteria 9](#_Toc512010120)

[5. Study Procedures 9](#_Toc512010121)

[5.1 Treatment Assignment 9](#_Toc512010122)

[5.2 Study Treatment 9](#_Toc512010123)

[6. Efficacy Assessments 10](#_Toc512010124)

[6.1 Definition 10](#_Toc512010125)

[6.2. Primary Endpoint 11](#_Toc512010126)

[6.3 Secondary Endpoints 11](#_Toc512010127)

[6.4 Methods and Schedule Of endpoints assessments 12](#_Toc512010128)

[7. Safety 12](#_Toc512010129)

[7.1 Safety Endpoints 12](#_Toc512010130)

[7.1.1 Clinical symptoms: 12](#_Toc512010131)

[7.1.2 Laboratory abnormalities: Incidence of adverse events, grade according to the CTCAE version3.0 13](#_Toc512010132)

[7.2 Adverse Events 13](#_Toc512010133)

[8. Rules of Follow-Up 13](#_Toc512010134)

[8.1 Follow-up Period 13](#_Toc512010135)

[8.2 Visit Scheduling 14](#_Toc512010136)

[8.3 Contents 14](#_Toc512010137)

[9. Data Analysis and Statistical Considerations 14](#_Toc512010138)

[9.1 Study Design Considerations 14](#_Toc512010139)

[9.1.1 Sample Size Assumptions 14](#_Toc512010140)

[9.1.2 Primary Efficacy Endpoint 15](#_Toc512010141)

[9.1.3 Secondary Efficacy Endpoints 15](#_Toc512010142)

[9.2 Data Analysis Considerations 15](#_Toc512010143)

[9.2.1 Analysis of efficacy 16](#_Toc512010144)

[9.2.2 Analysis of safety 16](#_Toc512010145)

[10. Materials For the Study 17](#_Toc512010146)

[11. Ethical Considerations 17](#_Toc512010147)

[11.1 Responsibility Of Investigators 17](#_Toc512010148)

[11.2 Informed Consent Process 17](#_Toc512010149)

[11.3 Protection of Subjects’ Personal Data 17](#_Toc512010150)

[12. Administrative Requirements 18](#_Toc512010151)

[13 Appendices 18](#_Toc512010152)

[13.1 Appendix 1 Definition and diagnosis of EBV-associated diseases 18](#_Toc512010153)

[13.2 Appendix 2 Definition and diagnosis of CMV-associated diseases 19](#_Toc512010154)

[13.3 Appendix 3 Diagnosis and Classification of aGVHD and cGVHD 19](#_Toc512010155)

# Protocol Summary

| applicant | Nanfang Hospital, Southern Medical University |
| --- | --- |
| phase | Ⅳ |
| indication | Patients with hematological malignancy who are scheduled to receive haploidentical hematopoietic stem cell transplantation (haplo-HSCT) |
| objectives | compare the incidence of viral infections and graft-versus-host disease (GVHD) in patients receiving haplo-HSCT between 7.5 mg and 10.0 mg ATG groups. |
| study design | prospective, multi-center, controlled, open-label, randomized phase Ⅳ study |
| required subject | subjects |
| Study sites | 4 (Nanfang Hospital, Peking University People's Hospital, Fujian Medical University Union Hospital, Xiangya Hospital) |
| Screening Criteria | subjects who are scheduled to receive haplo-HSCT without treatment contraindication |
| Control Group | 10.0 mg/kg ATG (2.5mg/kg/day from days -4 to -1 before transplantation) |
| Experimental Group | 7.5 mg/kg ATG (2.5mg/kg/day from days -3 to -1 before transplantation) |
| Primary Endpoint | Incidence of EBV DNAemia post-transplantation |
| Secondary Endpoints | incidence of grade II to IV and grade III to IV aGVHD within 100 days post-transplantation; hematopoietic engraftment; cGVHD; EBV-associated diseases; CMV infections (CMV DNAemia and associated diseases); relapse; non-relapse mortality (NRM); overall survival (OS); disease-free survival (DFS) and tolerability |
| Study procedures | This is a prospective, multi-center, controlled, open-label, randomized phase Ⅳ study. A total of 412 subjects with acute leukemia who were scheduled to receive haplo-HSCT and met the inclusion criteria is required. After giving written informed consent, all subjects will be randomly assigned to 7.5mg/kg (7.5 mg group) or 10.0mg/kg ATG group (10.0 mg group) in a 1:1 ratio. Viral infections (EBV and CMV), GVHD and survival will be evaluated. |
|  |  |

# 1. Background

Allogeneic hematopoietic stem cell transplantation (allo-HSCT) has been regarded as a curative strategy for acute leukemia. Lack of HLA-matched donor is the obstacle to widespread application of allo-HSCT. A growing body of clinical studies has suggested that haplo-identical related donor HSCT (haplo-HSCT) has comparable outcomes with transplants from matched sibling and unrelated donor, following the improvement of transplant technique in the last few years, such as immunosuppressive conditioning, graft manipulation, infection and graft-versus-host disease (GVHD) management. As a result, haplo-identical related donor is now considered an alternative for allo-HSCT. However, GVHD and infections remain main causes of morbidity and mortality after haplo-HSCT except leukemia relapse.

Now, the strategies of GVHD prophylaxis mainly included ex vivo and in vivo T-cell-depletion (TCD) in haplo-HSCT. Although ex vivo TCD could reduce the morbidity and mortality of GVHD, it also affects immune reconstitution leading to high incidences of infections and disease relapse, and so on, making less benefit for survival. Therefore, ex vivo TCD has been gradually replaced by in vivo TCD. Among the in vivo TCD strategies, two major methods are used: antithymocyteglobulin (ATG)/antilymphocyte globulin (ALG) or post-transplantation cyclophosphamide (PTCy). The ATG strategy has been widely used, especially in China, resulting in a declined infections and relapse compared with ex vivo TCD. Nevertheless, infections, especially viral infections, remain important drawback of ATG strategy. Several studies have demonstrated the risk of infections depends on the dosage of ATG. To data, the optimal dose of ATG which offers sufficient efficacy of GVHD prophylaxis and minimizes risk of infections is unknown in the haplo-identical setting. In our previous single-center study, 6mg/kg rabbit-ATG had a lower incidence of EBV infection, but higher risk of severe acute GVHD (aGVHD) and chronic GVHD (cGVHD) in haplo-HSCT, compared 10 mg/kg rabbit-ATG. Therefore, we design this multicenter randomized study to evaluate the effect of 7.5 mg/kg and 10 mg/kg rabbit-ATG aiming at lowering viral infections without increasing GVHD in haplo-HSCT.

# 2. Objectives

## 2.1 Primary Objective

The primary objective of this study is to compare the incidence of EBV DNAemia post-transplantation in patients receiving haplo-HSCT with 7.5 mg and 10.0 mg ATG for GVHD prophylaxis.

## 2.2 Secondary Objective

The secondary objective of this study is to compare the incidence of aGVHD and cGVHD, EBV-associated diseases, CMV infections (CMV DNAemia and associated diseases), relapse, survival and tolerance in patients receiving haplo-HSCT with 7.5 mg and 10.0 mg ATG for GVHD prophylaxis.

# 3. Study Design

This is a prospective, multi-center, controlled, open-label, randomized (1:1) phase Ⅳ study of comparison of different dosages ATG for GVHD prophylaxis. (7.5 mg/kg vs. 10.0 mg/kg) in patients receiving haplo-HSCT

Screening:

The subjects or their legally acceptable representative will provide written informed consent. EBV and CMV antibody will be performed in all the haplo-HSCT recipients and donors before transplantation. EBV and CMV-DNA was tested in all the recipients and donors.

Treatment:

Based on their assignment, subjects will receive 7.5 mg ATG (2.5mg/kg/day from days -3 to -1 before transplantation) or 10.0 mg ATG (2.5mg/kg/day from days -4 to -1 before transplantation).

# 4. Subject Selection Criteria

## 4.1 Subject Selection Criteria

### 4.1.1 Number of Subjects

A total of 412 subjects will be randomized to 7.5 mg group or 10.0 mg ATG group.

### 4.1.2 Inclusion Criteria

The subjects must meet the following criteria to be eligible for study entry:

1. Signed Informed Consent Form
2. Age≥14 years old and ≤65 years
3. Patients with acute leukemia
4. Ability to comply with study and follow-up procedures

### 4.1.3 Exclusion Criteria

1. Cardiac dysfunction (particularly congestive heart failure, ejection fraction <50%)
2. Respiratory failure ( PaO_2_ ≤60mmHg)
3. Hepatic abnormalities (bilirubin ≥3 mg/dL, aminotransferase >5 times the upper limit of normal)
4. Renal dysfunction (creatinine clearance rate < 30 mL/min)
5. ECOG performance status score 3 to 5
6. Uncontrolled infection
7. A history of anaphylaxis attributed to ATG
8. Subjects who have CMV- and EBV DNAemia or CMV and EBV associated diseases at transplantation
9. Subjects of whom the donor have CMV- and EBV DNAemia at transplantation

## 4.2. Removal Criteria

Subjects who have participated in the study will be removed from statistical analysis for any of the following:

1. Subjects who are ineligible for this study

2. Deviation(s) from the protocol

## 4.3. Withdrawal Criteria

Subjects will be withdrawn from study treatment for any of the following:

1. Inability to fully comply with the study protocol

2. Unacceptable toxicity

3. Patients refused to continue the study at any time after the randomization

4. Best interest of the subject based upon the investigator’s discretion

# 5. Study Procedures

## 5.1 Treatment Assignment

Patients who meet the eligibility criteria and consent to participate will be randomized at 1:1 ratio to receive 7.5 mg/kg ATG (7.5 mg group, experimental Group) or 10.0 mg/kg ATG (10.0 mg group, control group).

## 5.2 Study Treatment

ATG (Thymoglobulin; rabbit anti-human thymocyte immunoglobulin, Imtix Sangstat, Lyon, France) will be administered in 2.5mg/kg/day from days -3 to -1 in 7.5 mg group, and days -4 to -1 in 10.0 mg group. If ATG-related diarrhea or grade III fever (according to the Common Terminology Criteria for Adverse Events, the CTCAE, version3.0) occurred, additional dexamethasone (5.0 mg) would be given for treatment, and the speed of ATG infusion would be slowed down. If the subject experienced severe ATG associated allergic reaction at the first dose, he will be excluded from the study.

# 6. Efficacy Assessments

## 6.1 Definition

1. Hematopoietic engraftment was defined as the first of 3 consecutive days with an absolute neutrophil count exceeding 0.5×10^9^/L, and platelet reconstitution was defined the first day of a platelet count >20×10^9^/L without platelet transfusion Primary poor graft function (PGF) is defined as a slow or incomplete recovery of blood cell counts (ANC ≤0.5×10^9^/L and PLT ≤20×10^9^/L) by days +30 post-transplantation.

2. CMV viremia means positive CMV-DNA in the blood twice consecutively.

3. EBV viremia means positive EBV-DNA in the blood twice consecutively.

4. EBV-associated diseases are classified into EBV-associated post-transplant lymphoproliferative diseases (PTLD) and EBV-associated other diseases. The diagnosis of EBV-associated PTLD is according to the criteria of World Health Organization (WHO). The diagnosis of EBV-associated other diseases is based on the criteria of the European Conference on Infections in Leukemia and literatures, which include EBV-associated diseases with tissue other than lymphatic tissue involvement [see appendix 1].

5. The diagnosis of CMV-associated disease is according to the criteria of the European Conference on Infections in Leukemia [see appendix 2].

6. aGVHD is defined according to the 1994 Consensus Conference on Acute GVHD Grading and graded from I to IV grades, and cGVHD is graded as limited and extensive according to the literature [see appendix 3].

7. Relapse is defined as as hematologic relapse that were re-appearance of blasts in the peripheral blood, any manifestation of leukemia outside the hematopoietic system, or >5% blasts in the BM smear.

8. NRM is defined as death without relapse.

9. OS refers to survived until the final follow-up time point.

10. DFS is defined as survival in continuous CR without relapse.

11. Tolerability is evaluated based upon clinical symptoms and laboratory abnormalities (adverse events of ATG assessed according to the CACTE, version3.0). The clinical symptoms include the symptoms may be associated with the administration of ATG reported previously, including headache, arthralgia, bone pain, fatigue, fever, lightheadedness, petechiae, mucositis and diarrhea. The index of adverse events include hepatic function (ALT, AST, TBIL, DBIL) and renal function (BUN, Scr).

## 6.2. Primary Endpoint

Incidence of EBV DNAemia within 1 year post-transplantation

## 6.3 Secondary Endpoints

1. The incidences of grade II to IV and grade III to IV aGVHD within 100 days post-transplantation.

2. Hematopoietic engraftment

3. EBV-associated diseases

4. CMV infections (CMV DNAemia and associated diseases)

5. cGVHD

6. Relapse

7. NRM

8. OS

9. DFS

10. Tolerability

## 6.4 Methods and Schedule Of endpoints assessments

Patients will be followed for adverse events for 30 days after study treatment post-transplantation. The CMV-DNA and EBV-DNA loads of blood will be detected regularly by quantitative real-time polymerase chain reaction (RQ-PCR). The threshold for EBV-DNA and CMV-DNA copies in plasma provided by the manufacturer (ZJ Bio-Tech Co., Ltd., Shanghai, China) is less than 500 copies/ml. EBV-DNA or CMV-DNA is considered positive when the copies exceeded 500 copies/ml. The CMV-DNA and EBV-DNA of blood is monitored weekly for the first 3 months after transplantation; during the 4^th^ to 9^th^ month post-transplantation, the monitoring frequency was once every 2 weeks; and the frequency was once a month for the 10^th^ to 12^th^ month. If positive, viral load will be monitored twice a week. Endpoints assessments will be assessed according to the criteria previously mentioned throughout the study period.

# 7. Safety

## 7.1 Safety Endpoints

### 7.1.1 Clinical symptoms

The clinical symptoms include the symptoms may be associated with the administration of ATG reported previously.

 headache

 arthralgia

 bone pain

 fatigue

 fever

 lightheadedness

 petechiae

 mucositis

 diarrhea

### 7.1.2 Laboratory abnormalities: Incidence of adverse events, grade according to the CTCAE version3.0

 1. Hepatic function

 ALT

 AST

 TBIL

 DBIL

2. Renal function

 BUN

 Scr

## 7.2 Adverse Events

The investigator is required to make an assessment of the toxicity grade of each adverse events (AE) or serious adverse events (SAE) reported. In this protocol, the toxicity grade of each AE/SAE will be evaluated according to the CTCAE version 3.0.

# 8. Rules of Follow-Up

## 8.1 Follow-up Period

Starting after completion of ATG treatment or withdrawal from the study for other reasons.

## 8.2 Visit Scheduling

Every week for the first 3 months after transplantation, every two weeks during the 4th to 9th month post-transplantation, every month for the 10th to 24th month, and then every three months for the 25th to 36th month.

## 8.3 Contents

The contents of every follow-up visit include complaints of subjects, physical examination, hematology, urinalysis, biochemistry and viral loads in blood (EBV-DNA or CMV-DNA). All of the results must be documented in the original medical record.

# 9. Data Analysis and Statistical Considerations

## 9.1 Study Design Considerations

This is a prospective, multi-center, controlled, open-label, randomized (1:1) phase Ⅳ study of comparison of different dosages ATG for GVHD prophylaxis (7.5 mg/kg vs. 10.0 mg/kg) in patients receiving haplo-HSCT. The primary Endpoint was incidence of EBV DNAemia within 1 year post-transplantation, and the study is designed to determine if 7.5 mg/kg ATG is superior to 10.0 mg/kg in the study population with respect to EBV DNAemia.

### 9.1.1 Sample Size Assumptions

In the initial design, we predefined grade II to IV acute GVHD as the primary endpoint. We hypothesized that non-inferiority of 7.5 mg/kg ATG against 10.0 mg/kg ATG was established if the difference of the 95% CI in grade II to IV acute GVHD between the two groups was within 15%. On the assumption of an incidence of grade II to IV acute GVHD of 30%, an estimated 197 patients in each group were required at the one-sided significance level of 5% with a power of 90%. After the analysis of the first 210 patients enrolled in the study, the 100-day incidence of grade II to IV acute GVHD was 28.9% and 27.8% in the 7.5 mg/kg and 10.0 mg/kg groups with a difference of 1.2% with 95% CI of -10.0% to 13.4%. The 1-year incidence of EBV DNAemia in the 7.5 mg/kg group was significantly lower than that in the 10.0 mg/kg group (27.1% in 7.5 mg/kg group vs 45.2% in the 10.0 mg/kg group). According to the recommendation of the data and safety monitoring committee, the change of the primary endpoint was made to evaluate the superiority of a dose level of 7.5 mg ATG on viral infections without increasing aGVHD after haplo-HSCT.

The sample size calculation is with the following assumptions:

1. Incidence of EBV DNAemia within 1 year post-transplantation in 7.5 mg group: 30%
2. Incidence of EBV DNAemia within 1 year post-transplantation in 10.0 mg group: 42%
3. a 1:1 randomization scheme
4. a 5% 1-tailed risk of erroneously claiming a difference in the presence of no true underlying difference by Chi-square test or Fisher’s exact test
5. a 80% chance of successfully declaring a difference in the presence of a true underlying difference (power)
6. 5% percent of cases drop

Under the above assumptions, a total sample size of 412 subjects is required (206 in each group).

### 9.1.2 Primary Efficacy Endpoint

The primary efficacy endpoint was incidence of EBV DNAemia post-transplantation

### 9.1.3 Secondary Efficacy Endpoints

The incidence of grade II to IV and grade III to IV aGVHD within 100 days post-transplantation; hematopoietic engraftment; cGVHD; EBV-associated diseases; CMV infections (CMV DNAemia and associated diseases); relapse; non-relapse mortality (NRM); OS; DFS and tolerance.

## 9.2 Data Analysis Considerations

All collected data will be checked by two physicians for completeness and plausibility, and data analysis will be conducted according to the prespecified statistical analysis plan.

Descriptive summaries for continuous data will include the mean, standard deviation, median, minimum, and maximum. Descriptive summaries of discrete data will include the frequency and percentage of each category, as well as overall frequency and percentage totals.

### 9.2.1 Analysis of efficacy

For each numeric variable, the normality of distribution will be preliminarily assessed by the Kolmogorov-Smirnov test. Summary statistics, including proportions, mean, medians, and ranges, were used to describe the patient characteristics, pre-transplantation variables, adverse events, and immune recovery. The χ2 test and Mann-Whitney U test were used for categorical variables and continuous variables, respectively.

Time to acute GVHD, relapse, OS, and DFS were measured from the date of HSCT (d 0). OS and DFS were estimated using the Kaplan-Meier method. Considering the competing risks of death, cumulative incidence curves in a competing risks setting were used to calculate probabilities of aGVHD, cGVHD, viral infections (EBV- and CMV- emia as well as associated diseases) and relapse. The Cox proportional hazards model will be used to evaluate the associations of patient and transplant characteristics with outcomes in multivariate analysis. The risk factors for EBV and CMV infections include age and gender of patients; conditioning; EBV serostatus in patients and donors; CMV serostatus in patients and donors; ATG dose. The risk factors for aGVHD and cGVHD include age and gender of patients; conditioning; HLA disparity; age and gender of donors and ATG dose. The risk factors for survival and relapse include age and gender of patients; disease category; disease status; ATG dose.

### 9.2.2 Analysis of safety

All the patients in the study will be included in the safety analysis. The safety end points included clinical symptoms and laboratory abnormalities, laboratory values and vital signs analyzed by means of the chi-square test.

All P values are based on two-sided hypothesis tests. Alpha is set at 0.05. SPSS 19.0 (SPSS Inc., Chicago, IL, USA) and R version 3.4.3 (R Development Core Team, Vienna, Austria) were used for all data analysis.

# 10. Materials for the Study

All materials provided to study sites and investigators are as follows:

1. The study protocol
2. Informed consent
3. CRF

# 11. Ethical Considerations

## 11.1 Responsibility Of Investigators

The investigators have the responsibility for guarantee of the clinical study’s compliance with the protocol, Chinese good clinical practice (GCP) guidelines and applicable laws and regulations.

## 11.2 Informed Consent Process

Subjects must be informed about objectives, methods, possible benefits, potential risks and possible discomforts of the study by investigators before participation in the study. They should be informed that they can withdraw from the study at any time, and there is no impact on the treatment of the disease whether they take part in the study. Subjects or their legally acceptable representative should have enough time to read the inform consent and raise queries. Written informed consent must be obtained from each subject, or their legally acceptable representative.

## 11.3 Protection of Subjects’ Personal Data

Data collected in the study are limited to the efficacy and safety related to study treatment. Data will be collected and used in accordance with applicable laws and regulations.

# 12. Administrative Requirements

Any revisions of the protocol must be agreed by the investigator and the applicant. To insure the integrity, accuracy and reliability of the data, relevant results of examination and treatment must be documented in original medical record and CRF. Independent clinical monitoring was performed regularly by a panel of qualified and experienced study investigators composed of hematologists who are blinded as to the treatment assignments.

# 13 Appendices

## 13.1 Appendix 1 Definition and diagnosis of EBV-associated diseases

Swerdlow SH, Webber SA, Chadburn A, Ferry JA: Post-transplant lymphoproliferative disorders. World Health Organization Classification of Tumours of Haematopoietic and Lymphoid Tissues. Lyon: IARC Press; 2008:343–349.

Styczynski J, Reusser P, Einsele H, de la Camara R, Cordonnier C, Ward KN, Ljungman P, Engelhard D: Management of HSV, VZV and EBV infections in patients with hematological malignancies and after SCT: guidelines from the Second European Conference on Infections in Leukemia. Bone Marrow Transplant 2009, 43(10):757–770.

**Diagnostic criteria:**

Diagnosis of other EBV-associated diseases includes probable and proven EBV disease. Probable EBV disease: significant lymphadenopathy (or other endorgan disease) with high EBV blood load, in the absence of other etiologic factors or established diseases. Proven EBV disease (PTLD or other endorgan disease): EBV detected from an organ by biopsy or other invasive procedures with a test with appropriate sensitivity and specificity together with symptoms and/or signs from the affected organ. Diagnosis of PTLD must be based on symptoms and/or signs consistent with lymphoproliferative process developing after HSCT, together with detection of EBV by an appropriate method applied to a specimen from the involved tissue. Definitive diagnosis of EBV-PTLD requires biopsy and histological examination (including immunohistochemistry or flow cytometry for CD19+ and CD20+). EBV detection in biopsy specimen requires detection of viral antigens or in situ hybridization for the EBER (Epstein–Barr-encoded RNA) transcripts.

## 13.2 Appendix 2 Definition and diagnosis of CMV-associated diseases

Ljungman P, de la Camara R, Cordonnier C, Einsele H, Engelhard D, Reusser P, Styczynski J, Ward K: Management of CMV, HHV-6, HHV-7 and Kaposisarcoma herpesvirus (HHV-8) infections in patients with hematological malignancies and after SCT. Bone Marrow Transplant 2008, 42(4):227–240.

**Diagnostic criteria:**

CMV-associated disease is defined by the presence of clinical symptoms or signs of end organ disease, combined with the evidence of CMV infection in a tissue biopsy specimen. CMV pneumonia is diagnosed on the basis of signs and symptoms compatible with a diagnosis of pneumonia (hypoxemia, x-ray) and a bronchoalveolar lavage (BAL) fluid or lung biopsy specimen positive for CMV by immunohistology. CMV gastrointestinal (GI) disease is diagnosed when GI signs or symptoms occurred, and evidence of CMV in the GI tract was diagnosed by immunohistochemistry or in situ hybridization from biopsy specimens. CMV encephalitis is defined by the identification of central nervous system symptoms together with the detection of CMV-DNA in cerebrospinal fluid samples.

## 13.3 Appendix 3 Diagnosis and Classification of aGVHD and cGVHD

Przepiorka D, Weisdorf D, Martin P, et al. 1994 Consensus Conference on Acute GVHD Grading. Bone Marrow Transplant 1995;15:825-8.

Horwitz ME, Sullivan KM. Chronic graft-versus-host disease. BLOOD REV 2006;20:15-27.

**Grading of aGVHD**

| Grade | Degree of organ involvement |
| --- | --- |
| Ⅰ | Stage 1–2 skin rash; no gut involvement; no liver involvement; no decrease in clinical performance |
| Ⅱ | Stage 1–3 skin rash; stage 1 gut involvement or stage 1 liver involvement (or both); mild decrease in clinical performance |
| Ⅲ | Stage 2–3 skin rash; stage 2–3 gut involvement or 2–4 liver involvement (or both); marked decrease in clinical performance |
| Ⅳ | Similar to Grade III with stage 2–4 organ involvement and extreme decrease in clinical performance |

**Grading of cGVHD**

| Classification of cGVHD. | |
| --- | --- |
| Limited chronic GVHD | |
| Either or both: | |
| 1. Localized skin involvement | |
| 2. Hepatic dysfunction due to chronic GVHD | |
| Extensive chronic GVHD | |
| Either: | |
| 1. Generalized skin involvement, or | |
| 2. Localized skin involvement and/or hepatic dysfunction due to chronic GVHD | |
| Plus: | |
| 3a. Liver histology showing chronic aggressive hepatitis, bridging necrosis, or cirrhosis, or | |
| b. Involvement of eye (Schirmer test with <5-mm wetting), or | |
| c. Involvement of minor salivary glands or oral mucosa demonstrated on labial biopsy, or | |
| d. Involvement of any other target organ | |
