# Supplementary material for: Two dose levels of rabbit antithymocyte globulin as graft-versus-host disease prophylaxis in haploidentical stem cell transplantation: a multicenter randomized study
Source: BMC Med. 2019 Aug 12;17:156. doi: 10.1186/s12916-019-1393-7 (PMC6689871; doi:10.1186/s12916-019-1393-7)
Supplement: Supplementary file 2 — Table S1. Risk factors for aGVHD and cGVHD: Including univariate and multivariate risk factors analysis for aGVHD and cGVHD. Table S2. Immune reconstitution within 1 year posttransplantation (absolute value): Including the T, B, and NK cells reconstitution within 1 year posttransplantation shown by the absolute value. Table S3. Immune reconstitution within 1 year posttransplantation (percentage): Including the T, B, and NK cells reconstitution within 1 year posttransplantation shown by the percentage. Table S4. Causes of death posttransplantation. Table S5. Risk factors for survival and relapse: Including univariate and multivariate risk factors analysis for survival and relapse. (DOCX 51 kb) [file 12916_2019_1393_MOESM2_ESM.docx]

**Table S1. Risk factors for aGVHD and cGVHD**

| **Risk factors** | **Grade II to IV aGVHD** | |  | **Grade III to IV aGVHD** | |  | **cGVHD** | |  | **Extensive cGVHD** | |
| --- | --- | --- | --- | --- | --- | --- | --- | --- | --- | --- | --- |
|  | **Univariate** | **Multivariate**  **(HR, 95%CI)** |  | **Univariate** | **Multivariate**  **(HR, 95%CI)** |  | **Univariate** | **Multivariate**  **(HR, 95%CI)** |  | **Univariate** | **Multivariate**  **(HR, 95%CI)** |
| **Patient Gender,**  **male vs female** | 0.462 | 0.700  (0.92,0.61-1.39) |  | 0.887 | 0.660  (1.20,0.52-2.77) |  | 0.737 | 0.870  (0.97,0.68-1.39) |  | 0.080 | 0.150  (0.58,0.28-1.21) |
| **Patient age,**  **<27 y vs ≥27 y (median)** | 0.940 | 0.960  (0.99,0.67-1.47) |  | **0.094** | **0.038**  **(2.42,1.05-5.60)** |  | 0.321 | 0.360  (1.18,0.83-1.69) |  | 0.540 | 0.550  (0.83,0.44-1.55) |
| **Conditioning,**  **standard vs intensified** | 0.236 | 0.310  (1.25,0.81-1.93) |  | 0.769 | 0.840  (1.10,0.44-2.76) |  | 0.220 | 0.710  (1.08,0.71-1.65) |  | 0.077 | 0.150  (1.65,0.84-3.23) |
| **No. of HLA mismatched,**  **0-1 vs 2-3**  **0-1 vs 4-5** | 0.333  0.205 | 0.940  (1.04,0.42-2.54)  0.480  (1.36,0.59-3.14) |  | 0.415  0.609 | 0.780  (1.36,0.16-11.88)  0.620  (1.71,0.20-14.80) |  | 0.545  0.968 | 0.940  (1.03,0.49-2.16)  0.920  (1.04,0.51-2.10) |  | 0.752  0.848 | 0.900  (0.92,0.25-3.44)  0.970  (1.03,0.29-3.62) |
| **Donor gender,**  **male vs female** | 0.424 | 0.460  (1.17,0.77-1.79) |  | 0.862 | 0.760  (0.87,0.36-2.09) |  | 0.641 | 0.890  (1.03,0.70-1.50) |  | 0.160 | 0.230  (1.50,0.77-2.90) |
| **Donor age,**  **<30 y vs ≥30 y** | **0.016** | **0.028**  **(1.62,1.05-2.48)** |  | **0.004** | **0.003**  **(6.19,1.84-20.81)** |  | 0.214 | 0.300  (1.21,0.85-1.72) |  | 0.032 | 0.081  (1.80,0.93-3.48) |
| **ATG dose,**  **7.5 mg/kg vs 10.0 mg/kg** | 0.526 | 0.480  (0.875,0.60-1.27) |  | 0.321 | 0.510  (0.78,0.37-1.65) |  | 0.876 | 0.980  (1.00,0.71-1.41) |  | 0.402 | 0.370  (0.75,0.41-1.40) |

Abbreviation: GVHD, graft-versus-host disease; aGVHD, acute GVHD; cGVHD, chronic GVHD; HR, hazard ratio.

**Table S2. Immune reconstitution within 1 year posttransplantation (absolute value)**

|  |  | **CD3+** | |  | **CD3+CD4+** |  | **CD3+CD8+** |  | **CD19+** |  | **CD3-CD56+** |
| --- | --- | --- | --- | --- | --- | --- | --- | --- | --- | --- | --- |
| **Months after HSCT** |  | **Mean absolute value (10^9^/L, range)** | |  | **Mean absolute value (10^9^/L, range)** |  | **Mean absolute value (10^9^/L, range)** |  | **Mean absolute value (10^9^/L, range)** |  | **Mean absolute value (10^9^/L, range)** |
| **1^st^ (N=404)** | **7.5 mg/kg group** | 0.382  (0.004-1.198) | |  | 0.065  (0.001-0.229) |  | 0.128  (0.003-0.439) |  | 0.015  (0.000-0.121) |  | 0.525  (0.006-2.199) |
|  | **10.0 mg/kg group** | 0.858  (0.014-5.642) | |  | 0.100  (0.003-0.578) |  | 0.576  (0.006-3.919) |  | 0.017  (0.000-0.111) |  | 0.959  (0.009-3.226) |
|  | ***P*** | *0.560* | |  | *0.520* |  | *0.646* |  | *0.375* |  | *0.480* |
| **3^rd^ (N=312)** | **7.5 mg/kg group** | 2.075  (0.087-7.184) | |  | 0.189  (0.007-0.454) |  | 1.781  (0.079-6.365) |  | 0.119  (0.000-0.985) |  | 0.595  (0.006-1.800) |
|  | **10.0 mg/kg group** | 1.420  (0.036-3.784) | |  | 0.153  (0.006-0.696) |  | 0.745  (0.030-2.753) |  | 0.178  (0.000-1.334) |  | 0.297  (0.026-0.956) |
|  | ***P*** | *0.358* | |  | *0.612* |  | *0.079* |  | *0.651* |  | *0.156* |
| **6^th^ (N=275)** | **7.5 mg/kg group** | 3.157  (0.139-7.803) | |  | 0.830  (0.024-6.808) |  | 2.696  (0.108-6.875) |  | 0.185  (0.000-0.588) |  | 0.399  (0.014-1.072) |
|  | **10.0 mg/kg group** | 2.072  (0.042-6.086) | |  | 0.295  (0.006-0.974) |  | 1.321  (0.021-5.608) |  | 0.184  (0.000-0.933) |  | 0.405  (0.006-0.985) |
|  | ***P*** | *0.269* | |  | *0.306* |  | *0.098* |  | *0.988* |  | *0.970* |
| **9^th^ (N=252)** | **7.5 mg/kg group** | 4.227  (0.135-8.088) | |  | 0.591  (0.027-1.316) |  | 3.384  (0.101 -7.384) |  | 0.339  (0.016-1.141) |  | 0.556  (0.013-1.363) |
|  | **10.0 mg/kg group** | 4.779  (0.000-7.883) | |  | 0.777  (0.000-1.603) |  | 3.450  (0.000-6.092) |  | 0.387  (0.000-1.450) |  | 1.061  (0.004-2.583) |
|  | ***P*** | *0.642* | |  | *0.360* |  | *0.952* |  | *0.805* |  | *0.120* |
| **12^th^ (N=231)** | **7.5 mg/kg group** | 3.215  (0.431-6.763) | |  | 0.550  (0.046-1.211) |  | 2.162  (0.338-4.842) |  | 0.344  (0.036 -2.006) |  | 0.755  (0.008-2.931) |
|  | **10.0 mg/kg group** | 3.188  (0.264-7.434) |  |  | 0.426  (0.040-0.744) |  | 2.554  (0.218-6.498) |  | 0.441  (0.026-1.631) |  | 0.251  (0.055-0.531) |
|  | ***P*** | *0.980* |  |  | *0.460* |  | *0.636* |  | *0.708* |  | *0.148* |

**Table S3. Immune reconstitution within 1 year posttransplantation (percentage)**

|  |  | **CD3+** |  | **CD3+CD4+** |  | **CD3+CD8+** |  | **CD19+** |  | **CD3-CD56+** |
| --- | --- | --- | --- | --- | --- | --- | --- | --- | --- | --- |
| **Months after HSCT** |  | **Mean percentage (%, range)** |  | **Mean percentage (%, range)** |  | **Mean percentage (%, range)** |  | **Mean percentage (%, range)** |  | **Mean percentage (%, range)** |
| **1^st^ (N=404)** | **7.5 mg/kg group** | 31.53  (28.94-34.12) |  | 5.43  (5.32-5.53) |  | 5.97  (2.24-9.70) |  | 1.54  (0.14-2.93) |  | 54.16  (53.11-55.20) |
|  | **10.0 mg/kg group** | 39.25  (0.89-77.61) |  | 4.02  (0.08-7.95) |  | 27.18  (0.46-53.90) |  | 0.77  (0-1.53) |  | 53.65  (19.63-87.67) |
|  | **P** | 0.859 |  | 0.754 |  | 0.514 |  | 0.676 |  | 0.990 |
| **3^rd^ (N=312)** | **7.5 mg/kg group** | 68.77  (35.71-85.73) |  | 7.05  (3.19-15.05) |  | 58.91  (22.47-80.26) |  | 6.53  (0.12-37.16) |  | 23.31  (8.04-58.26) |
|  | **10.0 mg/kg group** | 78.06  (67.56-90.12) |  | 8.47  (2.86-17.08) |  | 39.00  (3.13-58.21) |  | 9.48  (0.19-35.96) |  | 12.26  (5.66-18.46) |
|  | **P** | 0.246 |  | 0.639 |  | 0.132 |  | 0.705 |  | 0.150 |
| **6^th^ (N=275)** | **7.5 mg/kg group** | 65.40  (2.92-91.79) |  | 21.10  (4.29-91.85) |  | 56.93  (14.71-77.45) |  | 3.22  (0-11.44) |  | 10.38  (2.18-25.48) |
|  | **10.0 mg/kg group** | 75.03  (60.65-84.41) |  | 11.43  (3.96-15.31) |  | 53.85  (31.07-77.78) |  | 7.71  (0-16.60) |  | 14.92  (10.01-19.67) |
|  | **P** | 0.476 |  | 0.451 |  | 0.780 |  | 0.103 |  | 0.241 |
| **9^th^ (N=252)** | **7.5 mg/kg group** | 79.37  (52.25-91.58) |  | 12.04  (5.49-21.79) |  | 61.99  (33.82-79.57) |  | 6.70  (0.93-18.37) |  | 10.81  (2.24-22.56) |
|  | **10.0 mg/kg group** | 76.07  (62.18-94.37) |  | 13.63  (3.98-31.01) |  | 52.52  (7.83-84.35) |  | 5.61  (0.07-13.09) |  | 18.40  (2.13-39.12) |
|  | **P** | 0.588 |  | 0.655 |  | 0.348 |  | 0.693 |  | 0.157 |
| **12^th^ (N=231)** | **7.5 mg/kg group** | 72.17  (59.72-85.52) |  | 13.02  (7.80-20.07) |  | 49.67  (17.04-59.61) |  | 6.90  (0.89-26.75) |  | 17.23  (6.00-39.08) |
|  | **10.0 mg/kg group** | 78.47  (63.96-92.58) |  | 12.31  (8.58-23.75) |  | 60.56  (43.75-80.92) |  | 11.61  (0.39-21.12) |  | 5.97  (2.76-10.03) |
|  | **P** | 0.249 |  | 0.800 |  | 0.136 |  | 0.267 |  | 0.036 |

**Table S4. Causes of death post-transplantation**

| **Cause of death** | **7.5mg group, N** | **10.0mg group, N** |
| --- | --- | --- |
| **Infections** | 15 | 20 |
| **Relapse** | 20 | 22 |
| **aGVHD** | 13 | 10 |
| **cGVHD** | 4 | 4 |
| **PTLD** | 0 | 2 |
| **Others**  **Thrombotic microangiopathy**  **Hemorrhage**  **PGF**  **Renal failure**  **Heart failure**  **Demyelinating disease**  **Unknown** | 10  2  2  1  0  0  1  4 | 14  2  3  1  1  1  0  6 |

Abbreviation: GVHD, graft-versus-host disease; aGVHD, acute GVHD; cGVHD, chronic GVHD; PTLD, posttransplant lymphoproliferative disorder; PGF, poor graft failure.

**Table S5. Risk factors for survival and relapse**

|  | **OS** | |  | **DFS** | |  | **Relapse** | |
| --- | --- | --- | --- | --- | --- | --- | --- | --- |
| **Risk factors** | **Univariate** | **Multivariate**  **(HR,95%CI)** |  | **Univariate** | **Multivariate**  **(HR,95%CI)** |  | **Univariate** | **Multivariate**  **(HR,95%CI)** |
| **Patient Gender,**  **Male vs Female** | 0.689 | 0.490  (0.87,0.59-1.29) |  | 0.976 | 0.750  (0.94,0.66-1.36) |  | 0.559 | 0.550  (1.18, 0.68-2.06) |
| **Patient Age,**  **<27 y vs ≥27y (median)** | 0.268 | 0.056  (1.41,0.99-2.00) |  | 0.247 | 0.060  (1.38,0.99-1.93) |  | 0.825 | 0.850  (1.05,0.64-1.71) |
| **Disease category,**  **AML vs ALL**  **AML vs ABL/ALAL** | 0.240  <0.001 | 0.030  (1.53,1.04-2.23)  0.002  (2.43,1.37-4.31) |  | 0.325  0.002 | 0.026  (1.50,1.05-2.16)  0.025  (1.97,1.09-3.56) |  | 0.098  0.165 | 0.350  (1.30,0.75-2.24)  0.400  (1.48,0.59-3.75) |
| **Disease status,**  **CR vs non-CR** | <0.001 | <0.001  (2.50,1.71-3.65) |  | <0.001 | <0.001  (2.82,1.97-4.03) |  | <0.001 | <0.001  (2.78,1.62-4.74) |
| **ATG dose,**  **7.5 mg/kg vs 10.0 mg/kg** | 0.308 | 0.400  (1.16,0.83-1.62) |  | 0.660 | 0.910  (1.02,0.74-1.39) |  | 0.546 | 0.250  (0.74,0.44-1.24) |

Abbreviation: HR, hazard ratio; AML, acute myeloid leukemia; ALL, acute lymphoblastic leukemia; ABL, acute biphenotypic leukaemia; ALAL, acute leukemia of ambiguous lineage; CR, complete remission.
